# Adaptation to Stimulus Statistics in the Perception and Neural Representation of Auditory Space

**DOI:** 10.1016/j.neuron.2010.05.018

**Published:** 2010-06-24

**Authors:** Johannes C. Dahmen, Peter Keating, Fernando R. Nodal, Andreas L. Schulz, Andrew J. King

**Affiliations:** 1 Department of Physiology, Anatomy and Genetics, Sherrington Building, University of Oxford, Parks Road, Oxford OX1 3PT, UK

**Keywords:** SYSNEURO, SYSBIO, SIGNALING

## Abstract

Sensory systems are known to adapt their coding strategies to the statistics of their environment, but little is still known about the perceptual implications of such adjustments. We investigated how auditory spatial processing adapts to stimulus statistics by presenting human listeners and anesthetized ferrets with noise sequences in which interaural level differences (ILD) rapidly fluctuated according to a Gaussian distribution. The mean of the distribution biased the perceived laterality of a subsequent stimulus, whereas the distribution's variance changed the listeners' spatial sensitivity. The responses of neurons in the inferior colliculus changed in line with these perceptual phenomena. Their ILD preference adjusted to match the stimulus distribution mean, resulting in large shifts in rate-ILD functions, while their gain adapted to the stimulus variance, producing pronounced changes in neural sensitivity. Our findings suggest that processing of auditory space is geared toward emphasizing relative spatial differences rather than the accurate representation of absolute position.

## Highlights

► Perceptual and neural adaptation were studied using near-identical paradigms ► Changing the variance of an acoustic signal affects perception ► Close correspondence between perceptual adaptation and ILD coding in the midbrain ► Coding of spatial cues seems to be geared toward representing relative differences

## Introduction

“A prime function of sensory centres is to code efficiently the patterns of excitation that occur, thus developing a less redundant representation of the environment” (Barlow, 1972). This statement was made in the context of a set of groundbreaking studies on the plasticity of the developing visual system (Blakemore and Cooper, 1970; Pettigrew and Freeman, 1973), which, together with subsequent studies in other sensory systems (Van der Loos and Woolsey, 1973; Zhang et al., 2001), established that the brain can, over the course of days to months, adapt to a modified sensory environment by altering the sensitivity of sensory neurons to more closely match the distribution of stimuli in that environment. Although it is generally assumed that this reallocation of resources confers a perceptual advantage, the only behavioral study to test this showed that expanded stimulus representations are associated with impaired sensory performance (Han et al., 2007). This underlines the importance of behavioral data in interpreting the impact of altered neural representations.

The brain's processing capacity is constrained not only by the number of neurons, but also by the number of spikes that each of them can generate over time. To use the neurons' limited dynamic ranges more efficiently, adjustments in coding strategy can be made throughout life and within seconds or milliseconds of encountering a change in the composition of the sensory input. For example, the recent history of stimulation can alter a neuron's response strength (Ulanovsky et al., 2003) and even the precision with which those stimuli are encoded so that the most frequently occurring values are encoded most precisely (Dean et al., 2005; Garcia-Lazaro et al., 2007; Watkins and Barbour, 2008; Wen et al., 2009).

A popular approach in investigating sensory adaptation is to manipulate the statistics of the entire distribution from which the stimuli are chosen. Although adaptation to higher-order stimulus statistics, such as skewness and kurtosis (Bonin et al., 2006; Kvale and Schreiner, 2004), or to the complex statistics of naturally occurring visual scenes (Sharpee et al., 2006) has been investigated, most studies have addressed this issue by changing the mean or variance of a stimulus ensemble. Adaptive coding has been examined most extensively in the visual system for changes in the statistics of light intensity fluctuations (Baccus and Meister, 2002; Chander and Chichilnisky, 2001; Dunn and Rieke, 2006; Mante et al., 2005; Smirnakis et al., 1997), but other visual stimulus dimensions (Brenner et al., 2000; Fairhall et al., 2001) as well as other sensory systems (Dean et al., 2005; Garcia-Lazaro et al., 2007; Kvale and Schreiner, 2004; Maravall et al., 2007; Nagel and Doupe, 2006) have also received attention.

The reported adjustments in neuronal responses are usually interpreted as providing a more efficient representation of the current stimulus environment. However, any evaluation of a change in coding strategy should also take into account its perceptual consequences and ask whether the change is behaviorally beneficial. So far, studies of that kind are lacking.

We chose to study perceptual and neural adaptation to stimulus statistics within the framework of auditory spatial processing, which is known to show considerable experience-dependent plasticity over various timescales (Furukawa et al., 2005; Kacelnik et al., 2006; Knudsen, 1983; Malone et al., 2002; Park et al., 2008; Phillips and Hall, 2005). Studying how spatial processing adapts to changes in input statistics is of particular interest because the accurate representation of absolute stimulus values—sound-source position in this case—may be more important than for other dimensions, such as light intensity processing, which relies more on the detection of relative differences in luminance across the visual scene.

A vivid percept of a stimulus changing position can readily be created over headphones by altering the levels of the signals delivered to the two ears in opposite directions. We presented human listeners and anesthetized ferrets with broadband noise sequences whose interaural level difference (ILD) rapidly fluctuated according to a Gaussian distribution, which creates the percept of a stimulus quickly taking new positions along the interaural axis. Changing the mean of the distribution shifts the range of possible positions to the left or right, while changing its variance widens or narrows the space of possible positions. We found that this led to corresponding adjustments in both the perception and neural representation of auditory space, and that the observed plasticity in neuronal response properties closely resembles the changes in perception.

## Results

### Perceptual Adaptation

**Figure 1:**
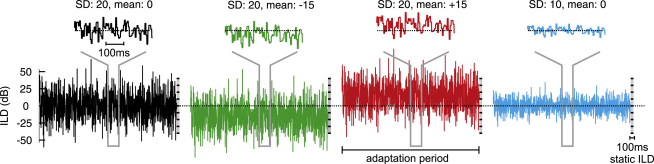
Stimulus Design Stimuli consisted of broadband noise presented at an average binaural level of 60 dB SPL, except where the level was fixed in the contralateral ear and varied on the ipsilateral side only. Zero ILD means that the sound level is equal in both ears; negative values, that it is higher in the contralateral ear (left ear for psychophysics). Trials/sweeps were presented in random order and consisted of an adaptation period with dynamically varying ILDs, immediately followed by a 100 ms long test stimulus with static ILD. During the adaptation period, a new ILD was drawn randomly every 5 ms from one of six Gaussian distributions. We used a “baseline” distribution with a mean of 0 dB and a standard deviation (SD) of 20 dB (shown in black), two distributions with shifted means (−15 dB: green, +15 dB: red), and three distributions with lower variances (SD = 10 dB: blue; SD = 15 dB and 5 dB, not shown). In psychophysical experiments, the adaptation period lasted 1 s and each subject was tested with a total of seven different static ILDs. In recording experiments, the adaptation period lasted 5 s, and nine values, evenly spread from −40 dB to +40 dB, were chosen for the static ILDs.

The human subjects indicated by a button press whether they perceived the position of the static stimulus presented immediately after the end of the 1 s adaptation period to be on the left or right of the midline, and their performance was characterized in terms of psychometric functions plotting the percentage of “left” responses as a function of ILD (see Figure 1 and Experimental Procedures for details of stimuli and analysis).

**Figure 2:**
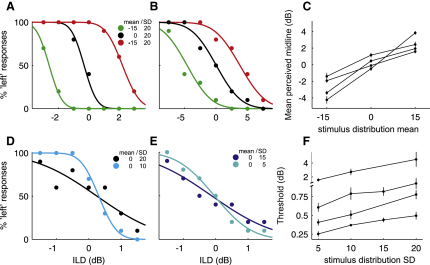
Human Psychophysics (A and B) Psychometric functions showing ILD sensitivity of two human listeners for distributions with different means. (C) Mean perceived midlines (dB) of four human listeners for distributions with different means. (D and E) Psychometric functions of two subjects for ILD distributions with different variances. (F) Mean thresholds (dB) for distributions with different variances from four subjects, two of whom were tested with four, and two with three, different variance conditions. Error bars are ± SEM.

Figures 2A and 2B show psychometric functions from two subjects following adaptation to ILD distributions with different means. The position of the functions clearly depends on the mean of the distribution. This was observed for all subjects tested, as shown in Figure 2C, which plots the mean perceived midline obtained using probit fits as a function of stimulus mean for each participant (Kruskal-Wallis results per subject: AN: n = 4, p = 0.0072; FS: n = 3, p = 0.027; JD: n = 8, p < 0.001; RC: n = 4, p = 0.0073). The subjects' perceived midline, where thresholds are expected to be lowest (Yost and Dye, 1988), therefore shifts in the direction of the ILD distribution they are adapted to. This corresponds to a shift in perceived stimulus position in the opposite direction. Consequently, stimuli are more likely to be perceived as being located to the left of the midline when presented in the context of a right-shifted stimulus distribution and vice versa.

Figures 2D and 2E show psychometric functions from two subjects following adaptation to ILD distributions with different variances. In both cases, the function from the lower-variance condition is steeper than that obtained with the higher variance. Such a relationship between stimulus variance and psychometric function slope was found for all subjects, as shown in Figure 2F, which plots the mean thresholds as a function of variance for each subject (Kruskal-Wallis: AN: n = 5, p = 0.018; JD: n = 7, p = 0.0016; RC: n = 7, p = 0.017; ST: n = 6, p = 0.0084). This suggests that, as stimulus variance decreases, perceptual sensitivity increases, allowing the correct lateralization of progressively smaller ILDs.

Our results show that the human auditory system adapts to a change in the mean of the ILD distribution, leading to a systematic bias and therefore a misjudgment of sound-source laterality, whereas adaptation to stimulus variance alters perceptual sensitivity. In order to understand what changes in coding strategy might bring about these perceptual effects, and whether this can be viewed as an effort to represent the sensory environment in the brain in the most “efficient” way, we measured neuronal responses in the ferret, a species that is widely used for studying auditory plasticity (Atiani et al., 2009; Bajo et al., 2010). We recorded from the inferior colliculus (IC) because previous studies have reported that substantial adaptation to changing stimulus statistics occurs in this nucleus (Dean et al., 2005; Kvale and Schreiner, 2004; Malmierca et al., 2009), while the ILD sensitivity of IC neurons can show pronounced shifts following cortical cooling (Nakamoto et al., 2008). The recordings were carried out under anesthesia in order to provide the stability needed for collecting complete data sets from individual neurons and to avoid changes in the arousal level or attentional modulation during the adaptation periods.

### Neuronal Adaptation

**Figure 3:**
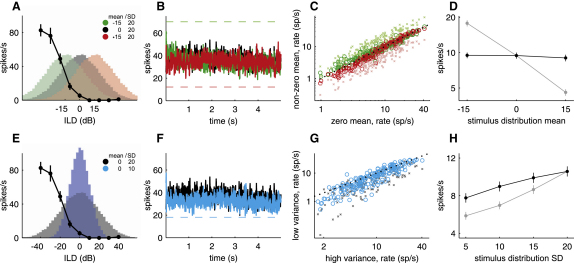
Spike Rate across Different Stimulus Distributions (A) Example baseline rate-ILD function of one neuron in the IC and histograms of three ILD distributions with different means. Each histogram shows the distribution of the 90,000 ILDs presented during all unique adaptation period sequences for a particular mean. (B) Average firing rate as a function of time (smoothed) from the same neuron for ILD distributions with different means, averaged over 90 unique adaptation periods. Dashed lines indicate expected adaptation rates based on the overlap between the ILD distribution and the baseline rate-ILD function (see Experimental Procedures for details on calculation of expected rates). (C) Observed adaptation rates during the baseline (zero mean) ILD distribution plotted against the observed (circles) and expected (crosses) adaptation rates for the distributions with mean ILDs of either −15 dB (green) or +15 dB (red). A circle lying on the black dotted unity line indicates that the adaptation rate remained constant across a change in mean. (D) Mean observed (black) and expected (gray) adaptation rate for distributions with different means. (E) Example baseline rate-ILD function for the same neuron whose response is shown in (A) and (B), together with histograms of stimulus distributions with two different variances. (F) Average firing rate as a function of time (smoothed) from this neuron for distributions with different variances. Dashed line indicates expected adaptation rate based on the overlap between the low-variance distribution and the baseline rate-ILD function. (G) Observed adaptation rates for the high- (SD = 20 dB) variance distribution versus observed (circles) and expected (crosses) adaptation rates for a low- (SD = 10 dB) variance distribution. A circle lying on the dotted black unity line indicates that the adaptation rate remained constant across a change in variance. (H) Mean observed (black) and expected (gray) adaptation rates for distributions with different variances. Error bars are ± SEM.

The simplest way to describe a neuron's behavior across different stimulus distributions is to count the number of spikes while presenting each distribution. The number of spikes fired by the neuron should be high when the distribution contains stimuli that mostly lie within its receptive field and lower when it contains fewer of those stimuli. The receptive field can, in the current context, be described by the neurons' rate-ILD function. Most IC neurons have monotonic rate-ILD functions, as illustrated by the baseline function obtained from an example neuron in Figures 3A and 3E. As the mean of the ILD distribution is varied (histograms in Figure 3A), the proportion of stimuli that overlap the function changes, so the number of spikes generated should change as well. However, the firing rate of this neuron during the 5 s adaptation periods showed a remarkable resistance to changes in the distribution mean, as shown by the almost identical rates during the adaptation periods for all three mean ILD values (Figure 3B). The rates during the negative and the positive distribution are initially slightly separated, but appear to converge and stabilize after a few hundred ms, suggesting that firing rates adjust quickly to the statistics of the distribution (see also Figure S1 available online). Although some neurons (red circles below and green circles above the unity line in Figure 3C) did change their mean firing rates during the adaptation period (“adaptation rate” hereafter) in the directions predicted from the position and shape of their baseline rate-ILD functions, these changes were usually smaller than expected, as illustrated by the greater distance between the unity line and the crosses, which indicate the expected adaptation rates. Similarly, the average adaptation rate for the entire population of neurons recorded did not shift as the mean of the distribution was changed (Figure 3D).

Because this neuron exhibited a rate-ILD function that was offset to the contralateral side (Figure 3E), decreasing the variance of the distribution would be expected to reduce the amount of overlap between its rate-ILD function and the stimulus distribution, thereby reducing its firing rate. This was not the case, however, because the neuron's adaptation rate remained almost identical across the two distributions (Figure 3F). The vast majority of IC neurons from which we recorded also had rate-ILD functions favoring the contralateral ear, but most, again, showed either no change or smaller than expected reductions when exposed to distributions with lower variances (Figures 3G and 3H).

These results indicate that when the stimulus distributions change, the neurons' output remains largely unaltered. Thus, the coding rules governing the translation of sensory input into spikes must change.

### Linear-Nonlinear Models

Linear-nonlinear models have had considerable success in capturing the computational changes associated with adaptation to stimulus statistics (Wark et al., 2007). These models characterize the coding process as initial linear filtering of the stimulus by the neuron, and output generation according to a nonlinear input-output function that relates the similarity between the stimulus and neuronal filter to spiking probability. We derived the linear filter by reverse correlating the stimulus sequences and the corresponding spike trains from the adaptation periods and extracting the spike-triggered average (STA). The stimulus was then passed through this filter (the time-reversed STA), resulting in a signal representing the similarity between stimulus and filter as a function of time. Using the corresponding spike trains, we then calculated the mean spike rate as a function of similarity to produce the input-output function. The STA provides an estimate of the stimulus feature that best drives a neuron. The filtered signal can be understood as a measure of how strongly that feature is represented in the stimulus. The input-output function characterizes the instantaneous relationship between the filtered signal and the neural response, and can be regarded as a measure of a neuron's sensitivity to its preferred stimulus feature. By describing the stimulus-response relationship and the stimulus-statistics-dependent changes in that relationship in terms of such a linear-nonlinear model, we can distinguish between adjustments in feature selectivity and neural sensitivity.

**Figure 4:**
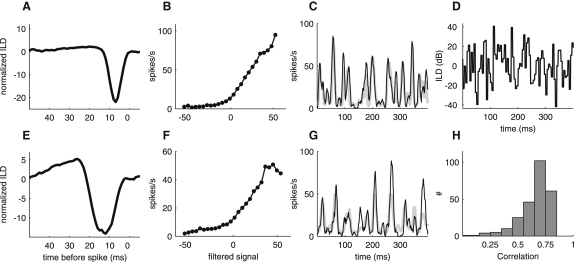
Linear-Nonlinear Models and Response Predictions for IC Neurons Derived from the Baseline Stimulus Distribution (A and E) The filter describes the stimulus feature that excites a given neuron, whereas the nonlinear input-output function (B and F) describes the sensitivity of the neuron to that feature. Most neurons exhibited largely monophasic filter shapes, such as these two examples, meaning that they were excited by negative deflections from the stimulus mean. (C and G) Recorded (averaged over 90 repeats) and predicted responses for these two neurons to the stimulus sequence shown in (D). A strong correspondence between recorded (thin dark line) and predicted (thick gray line) responses indicates that the linear-nonlinear model can successfully describe the relationship between stimulus and response. (H) Histogram of correlation coefficients between recorded and predicted responses for the whole sample of neurons in our study.

Figure 4 shows filters (Figures 4A and 4E) and input-output functions (Figures 4B and 4F) derived from the baseline ILD distribution for two IC neurons. Most filters were dominated by a large negative phase. Input-output functions were usually flat to start with, indicating that the similarity between stimulus and filter needs to pass a threshold before the neurons fire. This was followed by a monotonically rising component, signifying that the firing rate increases with growing similarity and then sometimes becomes saturated. To assess how well the linear-nonlinear models captured neuronal behavior, we used them to predict each unit's response to a stimulus sequence (Figure 4D) that was presented repeatedly, and compared the predicted response to the recorded one (Figures 4C and 4G). To avoid overfitting, this stimulus sequence was not used to estimate the models. The correlation coefficient was 0.83 for the unit in Figures 4A and 4C, at the upper end of the range for the whole population (Figure 4H), and was 0.69, identical to the population median, for the unit in Figures 4E and 4G. Thus, in most cases, the linear-nonlinear model provided a good description of the ILD sensitivity of the neurons. Units with correlation coefficients below 0.5 were excluded from further analysis of their filters and input-output functions.

### Linear-Nonlinear Models: Filter Shapes

**Figure 5:**
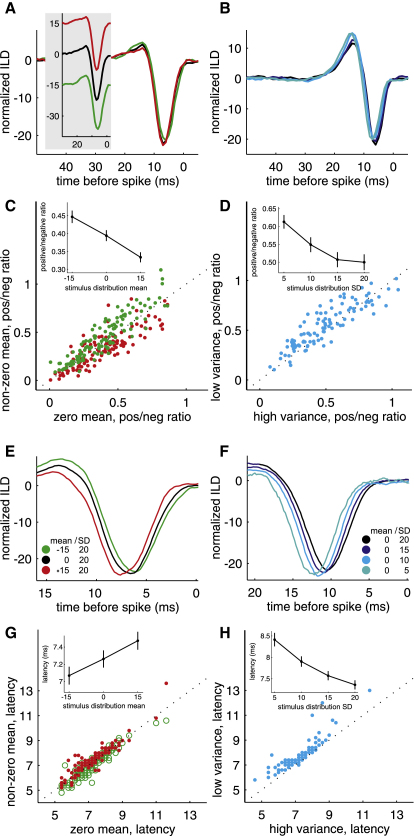
Effect of Varying the Mean and Variance of the Stimulus Distribution on Filter Shape and Time Course (A) Normalized filters derived for one IC neuron from stimulus distributions with different means. Insets show the same filters before mean subtraction. (B) Filters derived from stimulus distributions with different variances for a different neuron. (C) Ratio of positive to negative area (P/N ratio) for zero mean versus nonzero mean distributions. Inset shows mean P/N ratio for distributions with different means. (D) P/N ratio for high- (SD = 20 dB) versus low- (SD = 10 dB) variance distributions. Inset shows mean P/N ratio as a function of stimulus variance. (E) Filters derived for another neuron to show the effect of stimulus distributions with different means on latency (defined as the position of the negative peak). (F) Filters derived for a fourth neuron to show the effect of stimulus distributions with different variances on latency. (G) Filter latencies for zero mean versus nonzero mean distributions. Inset shows average filter latencies for distributions with different means. (H) Filter latencies for high- (SD = 20 dB) versus low- (SD = 10 dB) variance distributions. Inset shows mean filter latency as a function of variance. Although we used spike trains sampled at 5 kHz to analyze the time course of the filters, we often measured identical latencies for two or more neurons, resulting in many of the data points shown in the scatter plots of (G) and (H) occluding each other. Error bars are ± SEM.

Changes in stimulus mean or variance can induce large changes in filter shape and kinetics (Baccus and Meister, 2002; Bryant and Segundo, 1976; Mainen and Sejnowski, 1995; Nagel and Doupe, 2006). Here, we found that the filter shape of the neurons was very consistent across ILD distributions with different means, indicating that the neurons remained sensitive to the same stimulus features (Figure 5A). However, their preferred feature only remained the same relative to the current distribution's mean. This is shown by the inset in Figure 5A, which shows the same filters before mean subtraction. Thus, the ILD values that drive a neuron in the context of one distribution may be very different from those it is excited by following adaptation to a distribution with a different mean. For example, after adapting the neurons to an ILD distribution with a mean of +15 dB (red trace), presentation of a stimulus with an ILD of 0 dB represents a negative deflection from that mean, a feature that closely resembles their filters, and thus ought to excite the neurons. By contrast, the same stimulus value will be much less effective after adaptation to ILDs with a mean of 0 dB and particularly −15 dB.

Despite a high degree of similarity between filter shapes across different stimulus distributions, we did observe some small but systematic changes. Most filters had a large negative and a much smaller or no positive phase (Figures 5A and 5B) and therefore mostly behaved like integrators. The monophasic versus biphasic nature of the filters was quantified by the ratio of the positive to negative area (P/N ratio, Figures 5C and 5D). Shifting the mean of the ILD distribution toward a more negative value slightly changed that ratio in favor of the positive phase (Figures 5A and 5C, ANOVA: n = 118, p < 0.001). Reducing the variance of the distribution also produced a small increase in the P/N ratio and made the filters more biphasic (Figures 5B and 5D, ANOVA: n = 102, p < 0.001) and, thus, slightly more like differentiators, which may allow the neurons to pick out more stimulus contrast in a low-variance context. Although statistically significant at the population level, the average magnitude of the change in filter shape was small, closely resembling that illustrated by the example filters in Figures 5A and 5B.

Besides subtly influencing filter shape, we found that changing the mean or variance of the adaptation stimuli also affected the temporal relationship between stimulus and response. Filter latency, defined as the position of the negative peak, varied systematically with both mean and variance, as shown in the examples in Figures 5E and 5F. Changing the mean of the ILD distribution from −15 dB to +15 dB increased the latency by ∼0.4 ms (Figure 5G, ANOVA, n = 118, p = 0.019), whereas reducing the variance by a factor of four slowed down the time course of the filter by ∼1 ms (Figure 5H, ANOVA, n = 102, p < 0.001). Although these changes in time course are small and their functional consequences unclear, they fall within a range of timescales that is relevant to auditory processing. Thus, the timing of activity on the millisecond scale can guide plasticity in the auditory system (Dahmen et al., 2008; Tzounopoulos et al., 2004), while sensitivity to microsecond-scale interaural timing differences can be exploited to localize sound and may provide a basis for the processing of ILDs (Yin et al., 1985).

Interestingly, a reduction in stimulus variance produced an increase in both the P/N ratio and the filter latency. However, a negative shift in stimulus mean produced an increase in the P/N ratio, but a decrease in latency. This suggests that the mechanisms underlying changes in filter shape and time course may act independently from each other.

### Linear-Nonlinear Models: Input-Output Functions

**Figure 6:**
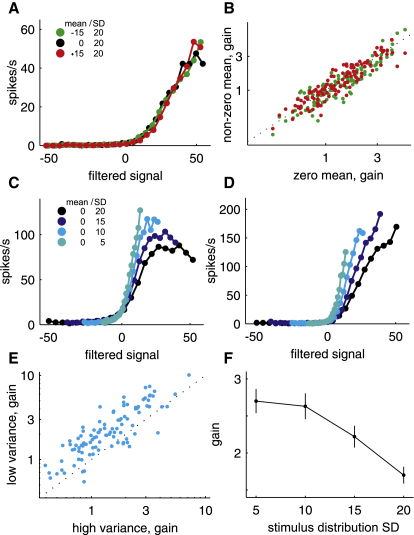
Gain of IC Neuron Input-Output Functions Depends on the Variance of the ILD Stimulus Distribution (A) Input-output functions for one neuron derived from stimulus distributions with different means. (B) Input-output function gains for zero mean versus nonzero mean distributions. (C and D) Input-output functions for two neurons derived from stimulus distributions with different variances. (E) Input-output function gain for high- (SD = 20 dB) versus low- (SD = 10 dB) variance distributions. (F) Mean input-output function gain for distributions with different variances. Error bars are ± SEM.

Figure 6A shows one unit's input-output functions for stimulus distributions with different means. Although there is some variation, we found no net change in gain across the population of neurons (Figure 6B, Kruskal-Wallis, n = 118, p = 0.386). Together with their largely stable feature preference, this should lead to neurons producing almost unchanged responses to very different stimuli as long as these have the same relationship to the mean of the distribution within which they occur. A different result was obtained when the variance was changed. Input-output functions became progressively steeper as the variance of the ILD distribution was reduced (Figures 6C and 6D). Such a relationship between neural gain and stimulus variance was observed in the vast majority of units (Figure 6E). On average, a 5 dB decrease in the standard deviation (SD) of the distribution increased the gain by ∼0.3 spikes/s per unit of filtered signal (Figure 6F, Kruskal-Wallis, n = 102, p < 0.001). This suggests that changing the variance, but not the mean, of the adaptation stimuli systematically alters the ILD sensitivity of the neurons. In contrast to other studies (Brenner et al., 2000; Fairhall et al., 2001), which used a more limited set of parameter values, the observed gain change was not linear over the entire range of values.

We varied ILDs by changing the sound level in each ear in opposing directions, thereby keeping the average binaural level constant, which is what happens naturally when sound-source location changes. However, we observed the same effects on ILD coding when we fixed the level in the contralateral ear and varied either the mean (Figure S2) or variance (Figure S3) on the ipsilateral side only.

### Rate-ILD Functions of IC Neurons

**Figure 7:**
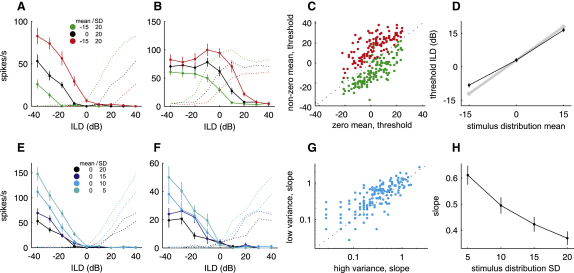
Rate-ILD Functions of IC Neurons (A and B) Rate-ILD functions for two neurons recorded in the left midbrain, for distributions with different means, and, as dotted lines, their hypothetical counterparts on the right side of the brain. (C) Thresholds for zero mean versus nonzero mean ILD distributions. (D) Black line shows the mean threshold for distributions with different means. Gray line indicates shifts in threshold equivalent in size to the 15 dB difference in the ILD distribution means. Rate-ILD functions with their baseline threshold <15 dB away from one end of the tested range were excluded from (C) and (D). (E and F) Rate-ILD functions for two neurons for distributions with different variances, and, as dotted lines, their hypothetical counterparts in the right IC. The functions in (A) and (E) belong to the same unit. (G) Mean slopes measured between ILDs of −20 dB and +20 dB for distributions with a high variance (SD = 20 dB) and those with a low variance (SD = 10 dB). (H) Mean slopes as a function of variance. Error bars are ± SEM.

From the response to the static ILDs presented at the end of each adaptation period, we constructed rate-ILD functions and examined whether these adapted to mean and variance manipulations of the preceding distribution in the way suggested by the linear-nonlinear models (see Figure S4 for an analysis of rate-ILD functions predicted from the linear-nonlinear models). Although adjustments in feature selectivity and neural sensitivity can only be distinguished using linear-nonlinear model analysis, the rate-ILD functions provide a ready means for measuring response variability as a function of ILD, and therefore allow us to investigate changes in coding precision in a straightforward fashion. The position of those functions clearly varied with the mean of the stimulus distribution (Figures 7A and 7B). To measure how far they are shifted in relation to each other, we measured their threshold ILDs. The unity line almost perfectly separates the stimulus conditions when each unit's zero-mean threshold ILD was plotted against the threshold ILDs derived with negative and positive means (Figure 7C). The difference between the mean threshold ILDs obtained with different adaptation stimuli was close to the 15 dB difference in the distribution means (Figure 7D, ANOVA, n = 144, p < 0.001), indicating that the neurons adapted almost perfectly to these changes.

Figures 7E and 7F show the rate-ILD functions of two neurons after adaptation to ILD distributions with four different variances. As expected, the slope of the rate-ILD functions changed with the variance of the stimulus distribution. These examples were typical of the population of IC neurons (Figure 7G), with the average slope of the functions increasing as the variance was reduced (Figure 7H, Kruskal-Wallis, n = 183, p < 0.001). Such changes in slope imply an increase in coding precision.

However, the slope alone may provide an inadequate measure of coding precision, because response variability ultimately limits the amount of information a neuron can convey. We therefore also calculated the standard separation *D* (Sakitt, 1973; Tollin et al., 2008), which takes both slope and response variability into account. *D* equals the difference in firing rate between adjacent ILDs divided by the geometric mean of their SDs. To obtain a continuous, smooth measure of *D*, we interpolated and smoothed the rate-ILD and SD-ILD functions.

**Figure 8:**
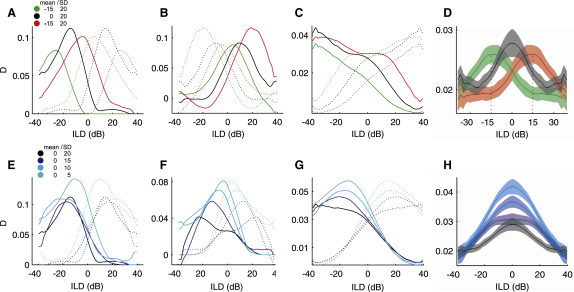
Changes in Coding Precision in the IC (A and B) Standard separation “*D*” (computed between adjacent values) for the rate-ILD functions shown in Figures 7A and 7B. (C) Mean standard separation *D* of the population of neurons from the left IC (solid lines) and the hypothetical population of neurons from the right IC (dotted lines) for distributions with different means. (D) Mean standard separation *D* of the combined left- and (hypothetical) right-IC population for distributions with different means. (E and F) Standard separation *D* for the rate-ILD functions shown in Figures 7E and 7F. (G) Mean standard separation *D* of the population of neurons from the left IC and the hypothetical population of neurons from the right IC (dotted lines) for distributions with different variances. The data in (C) and (G) were smoothed with a 20 dB boxcar function. (H) Mean standard separation *D* of the combined left- and (hypothetical) right-IC population for distributions with different variances. Shaded areas in (D) and (H) represent ± SEM.

The *D*-ILD functions (Figures 8A and 8D) shifted with the mean in the same way as the rate-ILD functions shown in Figures 7A and 7D. We recorded in the left IC only, but to get a full picture of how the neural changes might affect perception, we assumed that for each neuron in the left IC, there is one neuron on the right side that is a mirror-symmetric copy of the former. The black dotted lines in Figures 7A and 7B are such mirror-symmetric copies and the green and red dotted lines show how these neurons would behave when adapted to a distribution with a mean of −15 dB or +15 dB, respectively. The dotted lines in Figures 8A and 8B represent the *D*-ILD functions for these hypothetical neurons. Figure 8C shows the average *D*-ILD functions measured in the left IC and, as dotted lines, those corresponding to the hypothetical right IC, with the combined averages of both sides of the brain depicted in Figure 8D. The highest coding precision is found around the midline in the baseline condition, and shifts to an area centered on ∼−15 dB or ∼+15 dB when adapted to ILD distributions with means of −15 dB or +15 dB, respectively.

Figures 8E and 8F show *D*-ILD functions for the two example rate-ILD functions in Figures 7E and 7F (and as dotted lines for their hypothetical right-IC counterparts). In one case (Figures 7F and 8F), *D* increased in a way that matched the slope changes. However, the other example (Figures 7E and 8E) exhibited only relatively small increases in *D*, except in the lowest-variance condition, because response variability rose by a similar amount as the slope did. Nevertheless, on average, *D* went up as the stimulus variance decreased (Figure 8G), implying that response variability did not increase by as much as the rate-ILD function slopes. This therefore confirms that the population of IC neurons recorded in this study became more sensitive to ILDs as the variance of the stimulus distribution decreased. The combined averages of the two sides illustrate that the system as a whole exhibits enhanced coding precision over almost the full range of ILDs tested, but that the largest increases occur at the midline (Figure 8H).

## Discussion

To comprehend how and why sensory systems adapt to the composition of their environment, we require an understanding of how changes in input statistics affect both the perception and neural representation of that environment. With that in mind we designed a paradigm that allowed us to investigate how the perception of auditory spatial cues adapts to stimulus statistics and to characterize the underlying changes in neural coding.

We found that changing the mean of the ILD distribution shifted the perceived laterality of a stimulus presented within the context of that distribution away from the mean. This finding seems related to what is sometimes described as the “auditory localization after-effect,” which has been described in the free field as well as when binaural cues are presented over headphones (Carlile et al., 2001; Kashino and Nishida, 1998; Phillips and Hall, 2005). Although these studies differ in using a single static stimulus for the adaptor rather than dynamic stimuli whose values were chosen from a distribution, and tend to present adapters for a longer period of time, the perceptual shifts associated with changing the position of the adaptor are similar to the effects we observed when changing the mean of the entire distribution.

Altering the variance of the stimulus context also affected perception, with spatial sensitivity increasing as the variance of the stimulus distribution was decreased. To our knowledge, this is the first demonstration of how the variance of a dynamic acoustic signal affects perception.

We found that the responses of IC neurons change in the same way as the human psychometric functions. Thus, these neurons show corresponding adjustments in their rate-ILD functions when the distribution of the binaural spatial cues changes. In adapting to a change in the mean of the distribution, the neurons maintain their filter shape and adjust it to the current distribution's mean. As a result, and in keeping with the psychophysical data, they can respond almost identically to very different ILD values, as long as those stimuli have the same relationship to the mean of the distribution within which they occur. A change in stimulus variance alters coding strategy in a different way, with neuronal gain increasing as the variance of the stimulus distribution goes down. This enables the same change in input to be represented by a larger change in firing rate in a low-variance environment, which corresponds to the higher perceptual sensitivity exhibited by human listeners when lateralizing stimuli under these conditions.

An important consequence of mean-dependent changes in stimulus coding is that the neurons are ill equipped to provide information about the absolute ILD of a stimulus, and therefore its position in space. This will result in systematic misperceptions, as observed in our psychophysical data. However, the fact that the area of highest coding precision shifts almost perfectly with the mean of the distribution may provide an explanation for these effects. Thus, the brain attempts—at the cost of an ability to judge absolute stimulus position—to maintain the highest perceptual sensitivity in that area of space where most of the stimuli occur. For a Gaussian distribution, this area changes with the mean. Assuming that the mechanisms underlying adaptation to static stimuli are related to the mean-dependent changes we see with a dynamic signal, several studies showing that perceptual sensitivity can improve when adaptor and target sounds occupy similar azimuthal locations (Getzmann, 2004) or have similar binaural cue values (Kashino, 1998; Maier et al., 2009; Sach et al., 2000) seem to support the notion that spatial sensitivity does indeed shift as a function of stimulus mean.

The smaller range of stimuli that needs to be encoded in a low-variance context allows neural gain to be increased, which improves coding precision and provides a possible basis for enhancing human ILD sensitivity. The capacity of the auditory system to adapt to stimulus variance by changes in gain may also have a cost though, because this should increase the perceived distance between two stimuli in a low-variance context and decrease it in a high-variance context and, effectively, stretch and compress auditory space. In a free-field localization task, such a distortion of auditory space would be expected to result in a systematic overshooting of absolute location judgments (i.e., distance from midline) in a low- relative to a high-variance context, and systematic undershooting in a high- compared with a low-variance context.

With the caveat that our study investigated the processing of just one sound localization cue, the nature of the adjustments to input statistics that we described suggests that the processing of auditory space is geared toward the representation of relative positional differences between stimuli, rather than their absolute positions in space. This resembles the specialization for relative disparity in the binocular processing of visual depth (Thomas et al., 2002). Such a coding strategy may also account for why it has not been possible to find a map of auditory space within the brain other than at the level of the superior colliculus (King and Hutchings, 1987; Middlebrooks and Knudsen, 1984) and its midbrain sources of auditory input (Binns et al., 1992; Schnupp and King, 1997), where auditory and other sensory inputs are used to direct orienting responses.

We observed a very close similarity between the effects of changing the input statistics on human ILD lateralization and on the responses of neurons recorded in the IC of anesthetized ferrets. This implies that adaptation to stimulus statistics, which has previously been described in isolated retinae (Baccus and Meister, 2002; Chander and Chichilnisky, 2001; Kim and Rieke, 2001; Smirnakis et al., 1997), insects (Brenner et al., 2000; Fairhall et al., 2001), and both anesthetized (Bonin et al., 2006; Dean et al., 2005, 2008; Garcia-Lazaro et al., 2007; Kvale and Schreiner, 2004; Mante et al., 2005; Maravall et al., 2007; Sharpee et al., 2006; Wen et al., 2009) and awake (Nagel and Doupe, 2006; Watkins and Barbour, 2008) vertebrates, can occur at a relatively early stage of processing, and that the associated changes in perceptual sensitivity can largely be accounted for without having to invoke higher-level, task-dependent effects. Given that ILDs represent differences in sound level at the two ears, any adjustment in level coding in monaural brainstem pathways could also influence ILD sensitivity. Consequently, adaptation to sound level occurring as early as the auditory nerve (Wen et al., 2009) might affect the neural processing and perception of ILDs. Nevertheless, given recent evidence for corticofugal modulation of auditory spatial processing (Bajo et al., 2010; Nakamoto et al., 2008), it could be the case that descending projections to the IC contribute to the adjustments observed in the current study.

Candidate mechanisms for adaptation to stimulus statistics include GABA release from lateral superior olive neurons onto presynaptic GABA_B_ receptors, which has been shown to induce shifts in the rate-ILD functions of these neurons and affect their slopes (Magnusson et al., 2008). In the somatosensory system, calcium-dependent, slow afterhyperpolarization is associated with variance-dependent changes in gain (Díaz-Quesada and Maravall, 2008), whereas gain adjustment in retinal ganglion cells is mediated through sodium current modulations (Kim and Rieke, 2003). Given the speed with which changes in coding such as those we observed can occur (Baccus and Meister, 2002; Brenner et al., 2000; Fairhall et al., 2001; Nagel and Doupe, 2006), it has been suggested that they may be better accounted for by fixed nonlinearities rather than by time-dependent alterations in stimulus-response relationships (Borst et al., 2005). A detailed investigation into the time course of adaptation at both perceptual and neuronal levels should allow us to constrain these potential mechanisms.

## Experimental Procedures

All electrophysiological and psychophysical experiments were carried out in sound-attenuated chambers (Industrial Acoustics Company, Winchester, UK), and were approved by the relevant local ethical review committees, and, in the case of the ferret recording experiments, licensed by the UK Home Office.

### Psychophysics

We tested a total of five human adults (two males, three females), four of whom were naive to the purpose of the study. Four of these subjects took part in the mean-adaptation task, and four in the variance-adaptation task. Matlab (The MathWorks, Natick, MA) was used to control stimulus presentation and response collection and for data analysis. Stimuli were generated using TDT System 3 processors (Tucker Davis Technologies, Alachua, FL) and were presented over headphones (Sennheiser HD25, Wedemark-Wennebostel, Germany). Subjects responded by button presses on a keyboard.

### Electrophysiological Recording

Five adult ferrets were used in this study. Animals were sedated with an i.m. injection of medetomidine hydrochloride (Domitor; Pfizer Ltd., Walton Oaks, UK) and, after insertion of an i.v. cannula, maintained under anesthesia with continuous infusions of Domitor (22 μg/kg/hr) and ketamine hydrochloride (5 mg/kg/hr, Ketaset; Fort Dodge Animal Health Ltd., Southampton, UK) in a 0.9% saline solution supplemented with 5% glucose. A single s.c. dose of 0.06 mg/kg/hr atropine sulfate (C-Vet Veterinary Products, Leyland, UK) was provided, along with 0.5 mg/kg dexamethasone (Dexadreson; Intervet UK Ltd., Milton Keynes, UK) about every 12 hr, in order to reduce the risk of bronchial secretions and cerebral edema, respectively. The animals were intubated and artificially ventilated with oxygen. End-tidal CO_2_ and heart rate were monitored and body temperature was maintained at 38°C using a rectal probe coupled to a heating blanket.

The skull was exposed and a stainless steel bar was attached with screws and dental cement above the right hemisphere. A craniotomy was made over the left lateral gyrus, the dura was removed, and silicone oil was applied to protect the cortical surface. A single-shank silicon probe electrode (Neuronexus Technologies, Ann Arbor, MI) with 16 recording sites spread over a length of 1.5 mm was lowered through the cortex into the central nucleus of the IC. The position of the probe was confirmed by inspection of the units' frequency response areas and by the existence of the characteristic dorsoventral tonotopic gradient. Stimuli were presented through a pair of earphones (Panasonic, RP-HV298, Bracknell, UK) attached to otoscope speculae that were inserted into each ear canal.

Neural signals were band-pass filtered (500 Hz – 3 kHz), amplified, and digitized (25 kHz) using TDT System 3 processors. Matlab and BrainWare (Tucker Davis Technologies) were used to control stimulus presentation and data acquisition, action potential clusters were extracted in BrainWare, and all further data analysis was carried out in Matlab.

### Stimuli

Stimuli consisted of broadband noise presented at an average binaural level of 60 dB sound pressure level (SPL). Zero ILD means that the level is equal in both ears; negative values, that it is higher in the contralateral ear (left ear for psychophysics). Trials/sweeps were presented in random order and consisted of an adaptation period with dynamically varying ILDs, immediately followed by a 100 ms long test stimulus with static ILD. During the adaptation period a new ILD was drawn randomly every 5 ms from one of six Gaussian distributions. These comprised a baseline distribution with a mean of 0 dB and an SD of 20 dB, two distributions with shifted means (−15 dB and +15 dB), and three distributions with reduced variances (SD = 5, 10, and 15 dB).

For psychophysical measurements, we used relatively short, 1 s long adaptation periods, in order to minimize the total amount of time required for testing. Each participant was tested with a total of seven different static ILDs, chosen according to individual acuity. Each session consisted of at least 210 trials and yielded psychometric functions for at least three different distributions (70 trials per distribution). For each subject, we collected three to eight psychometric functions per distribution.

For electrophysiological experiments, it was desirable to have longer adaptation periods because the initial part of the data from each adaptation period sequence, equivalent in length to the duration over which the STA was calculated, had to be discarded. We therefore used 5 s durations. Pilot experiments indicate that neural and perceptual adaptation to a new distribution is complete after a few 100 ms, suggesting that this difference in the length of the adaptation period has no consequences for the results reported here. Nine values, evenly spread from −40 dB to +40 dB, were chosen for the static ILDs. For each distribution, 180 sweeps were presented in random order. In 90 of these sweeps, the same sequence was used for the adaptation period (hereafter referred to as “repeated sequences”). In the other 90 sweeps, each adaptation period consisted of a different sequence (hereafter referred to as “unique sequences”). This was done so that the linear-nonlinear models could be fitted and tested using different data sets.

### Data Analysis

The probit method was used to fit psychometric functions to the data from the human subjects, from which the perceived midline (ILD value associated with 50% correct responses) and the threshold (difference between 50% and 75% ILD) were extracted.

Spike sorting was performed offline using an automated k-means clustering algorithm in BrainWare. Spike clusters that exhibited a clear refractory period in the autocorrelation histogram were classed as single units, and all others were classed as multiunit clusters. Separate analyses did not reveal differences between single units (n = 126) and multiunit clusters (n = 129) in our data set, and these were therefore combined.

All 255 units analyzed exhibited a monotonic relationship between firing rate and ILD. The few additional units encountered with nonmonotonic (peaked or u-shaped) response functions (n = 21) were not included in the data set. The characteristic frequencies of the neurons ranged over almost six octaves, but no association could be detected between frequency selectivity and any of the aspects of mean or variance adaptation that we investigated.

Rate-ILD function refers to the mean spike rate per second as a function of ILD, measured 5–50 ms after the onset of the static ILDs. Adaptation rate is the recorded mean spike rate over the adaptation period, averaged across all adaptation periods of the 90 sweeps with unique sequences. Expected adaptation rate (*eRate*) refers to the firing rate that a neuron is expected to produce during nonbaseline stimulus distributions under the assumption that it is entirely dependent on the shape and position of the baseline rate-ILD function. This was calculated by taking the dot product of the baseline rate-ILD function (*bRIF*) and a given stimulus distribution (*Dist)*. The result was normalized by the dot product of the baseline rate-ILD function and the baseline distribution (*bDist*) and scaled by the baseline adaptation rate (*bRate*) so that it could be expressed in spikes/s.

$$eRate=\frac{bRIF•Dist}{bRIF•bDist}\times bRate$$

For this purpose, each distribution, consisting of the stimulus values presented during the 90 unique adaptation sequences, was expressed as a vector representing the distribution as a histogram with 1 dB resolution (the histograms in Figures 3A and 3E have a resolution of 2.5 dB). The baseline rate-ILD functions were, therefore, interpolated at a resolution of 1 dB. Because the flanks of the distributions went beyond the range of the rate-ILD functions, these were extended to cover the same range as the distributions by padding their negative and positive ends with the values obtained for −40 dB and +40 dB, respectively.

For estimation of the linear-nonlinear models [see Figure S5 and Chichilnisky (2001), Baccus and Meister (2002), and Simoncelli et al. (2004) for more detailed descriptions], only data from the unique stimulus sequences were used. The linear filter represents the time-reversed STA of the mean-subtracted signal. This was computed by summing all stimulus waveforms preceding a spike and dividing by the total number of spikes. STAs were calculated over a length of 100 ms after ignoring the first 100 ms of each trial. Before passing the mean-subtracted stimulus sequences through the filter, the filter was normalized (Baccus and Meister, 2002; Fairhall et al., 2001) so that the variance of the stimulus was equal to the variance of the filtered stimulus. This normalization ensured that an observed change in gain was not due to a change in filter amplitude. The range of filtered stimulus values was then divided into 40 bins of even size. By relating the filtered stimulus sequences to the corresponding spike trains, we computed the average firing rate associated with each bin and plotted these firing rates against the bin centers to produce the input-output function. Bins with fewer than 500 counts were not included.

To evaluate the model's accuracy, we predicted responses to the repeated stimulus sequence. Different data sets were used to fit (data from unique sequence) and test (data from repeated sequences) the models, in order to avoid overfitting. The stimulus sequence was passed through the filter, and the filtered signal was then transformed into firing rates according to the input-output function. Before calculation of the correlation coefficient between predicted and recorded responses, the average response to the 90 repeated stimulus sequences was smoothed with a 5 ms boxcar window. Because some neurons showed a gradual adjustment in firing rate over the first 500 ms of the sequence, the reported correlation coefficients are based on calculations that omitted this initial segment, although its inclusion mostly had relatively little impact on the obtained coefficients. Note also that uncorrected response-prediction correlation coefficients can be subject to an underestimation bias (Petersen et al., 2008); even if the LN model of a unit was perfect, the noise introduced through finite sampling of the PSTH would cause the correlation coefficient to be <1.

The P/N ratio was calculated by dividing a filter's positive area by its negative area. Filter latency was calculated as the distance between the negative peak and 0. For this part of the analysis, we used spike trains sampled at 5 kHz instead of 1 kHz. The gain of the input-output function was calculated as the average slope of the function, excluding subthreshold and saturation regions [defined as regions whose slope was less than 5% of the maximum slope (Nagel and Doupe, 2006)], and was expressed as spikes/s per unit of filtered signal.

The threshold of each rate-ILD function was defined as the point at which the firing rate exceeded the unit's minimum rate by 5% of the unit's maximum rate. For this purpose, the functions were interpolated at a resolution of 1 dB and smoothed with a 20 dB wide boxcar function. The slope of the rate-ILD functions was measured as the average slope between −20 dB and +20 dB and was expressed in spikes/s per dB. The standard separation *D* between two stimulus values is equal to their difference in firing rate divided by the geometric mean of their SDs. For Gaussian random variables, *D* is equal to the Fisher information, another popular measure of coding precision. In practice, standard separation and Fisher information also tend to converge on similar results (Wen et al., 2009). One-way ANOVA was used to test for statistically significant differences between experimental conditions unless variables were not normally distributed or sample sizes were very small, in which case the Kruskal-Wallis test was used instead.
