# Supplementary material for: Adaptation to Stimulus Statistics in the Perception and Neural Representation of Auditory Space
Source: Neuron. 2010 Jun 24;66(6):937–48. doi: 10.1016/j.neuron.2010.05.018 (PMC2938477; doi:10.1016/j.neuron.2010.05.018)
Supplement: Document S1. Supplemental Figures [file mmc1.pdf]

Supplemental Data: Adaptation to stimulus statistics in the perception and neural representation of auditory space

Johannes C Dahmen, Peter Keating, Fernando R Nodal, Andreas Schulz and Andrew J King

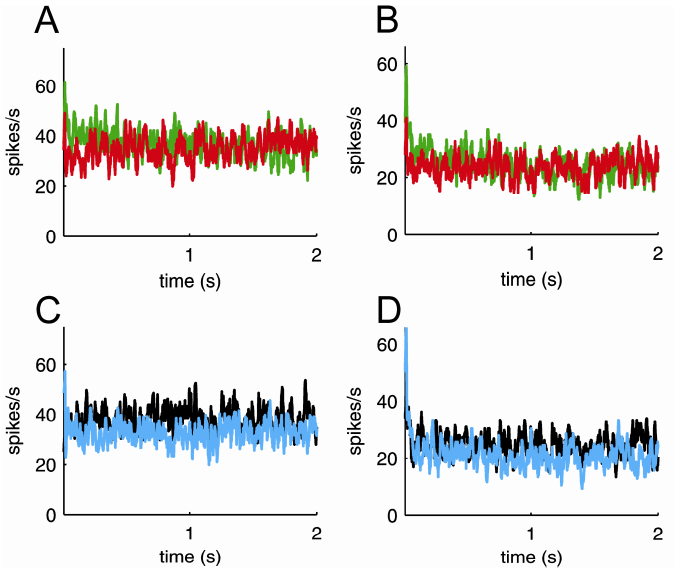

Figure S1. Spike rates adjust rapidly. Related to Figure 3.
(A) Same as Figure 3B, except that only the PSTHs of the two mean-shifted distributions are plotted and only the first 2000 ms are shown, as this better illustrates the rapid adjustment in firing rate taking place. Initially the firing rates differ slightly but they have converged and reached a stable state by ~500 ms after the onset of the stimulus sequences. (C) First 2000 ms of PSTHs from Figure 3F. Here the adaptation rates reach a stable state almost immediately after the onset of the stimulus sequences. The same applies to the PSTHs plotted in (B) and (D). The rate-ILD functions of the neuron whose responses are plotted in (B) and (D) can be seen in Figure 7A and E. Although these data give some indication of the dynamics of the observed adaptation, it should be noted that they show how neurons adjust from silence (i.e. following a gap of ~1 s between trials) to a given stimulus distribution. The more interesting question is how fast neural representations (and perceptions) adapt from the statistics of one distribution to the statistics of a different one. This requires a slightly different paradigm and is the focus of current efforts in our lab.

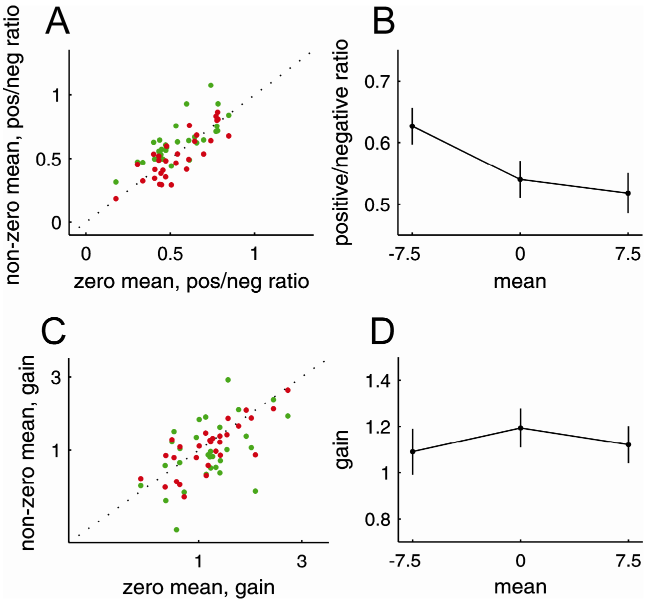

Figure S2. Effects of varying the mean of the ILD distribution on filter shape and gain. Related to Figures 5 and 6.

In this case, ILDs were generated by modulating the sound level at the ipsilateral ear and holding the level constant at the other ear. Note that holding the level constant in one ear reduces the ILDs by half. The means are, therefore, -7.5, 0 and +7.5 dB. A subtle change in filter shape (A and B, Kruskal-Wallis, n = 30, P = 0.036) and no systematic change in gain (C and D, Kruskal-Wallis, n = 30, P = 0.46) was observed when the ILD distribution mean was varied. The same happened when ILDs were generated by varying the level at each ear in opposing directions (Figures 5 and 6). Error bars are ± one SEM.

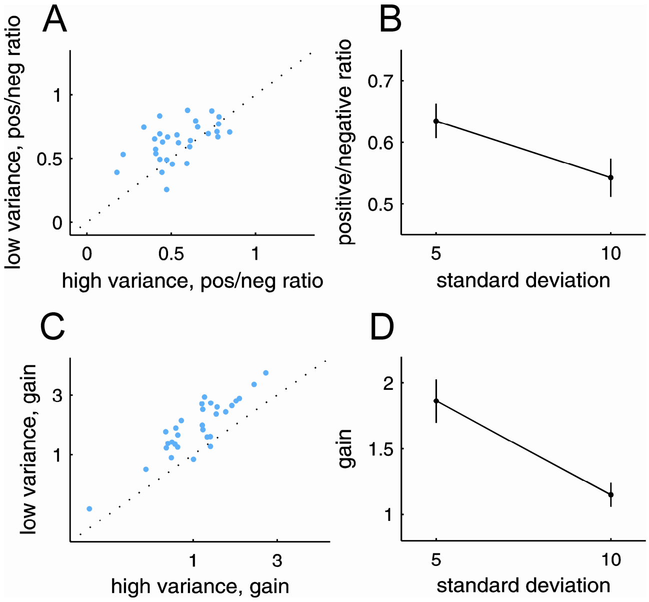

Figure S3. Effects of changing the variance of the ILD distribution on filter shape and gain. Related to Figures 5 and 6.

In this case, ILDs were generated by modulating the sound level at the ipsilateral ear and holding the level constant at the other ear. Neurons were tested on distributions with two different variances (SD = 5 and SD = 10). In Figures 5 and 6, we showed that changing the variance of the ILD distribution by varying the sound level in both ears has only a subtle effect on filter shape, but substantially changes neuronal gain. Neurons, thus, largely retain their feature preference, but become more sensitive when the variance of the stimulus distribution is reduced. The same effects on filter shape (A and B, Wilcoxon rank sum test, n = 30, P = 0.035) and neural sensitivity (C and D, Wilcoxon rank sum test, n = 30, P < 0.001) were observed when ILDs were generated by modulating the level in the ipsilateral ear alone. Error bars are ± one SEM.

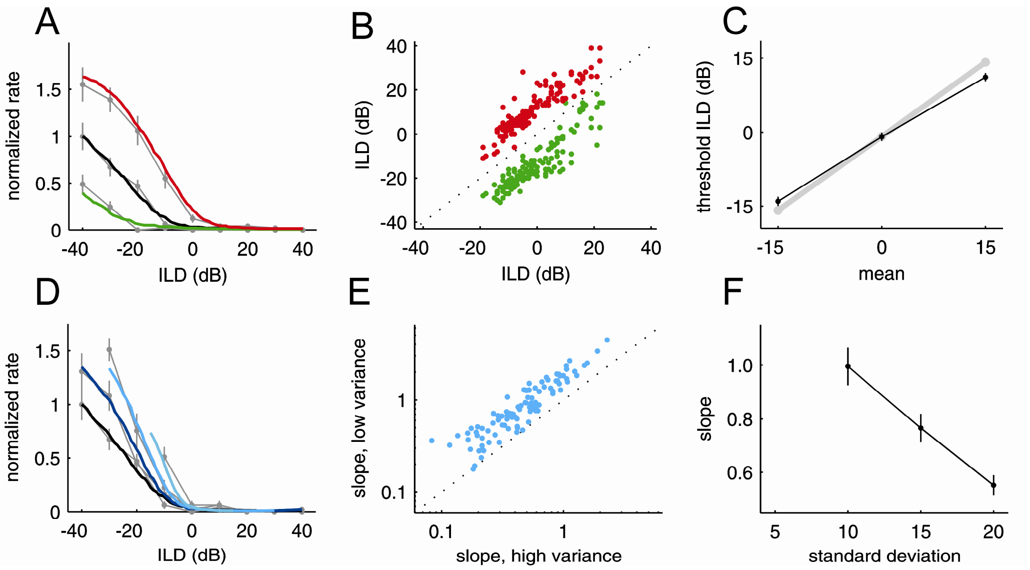

Figure S4. Rate-ILD functions predicted from linear-nonlinear models correctly anticipate the effects of mean and variance on the recorded rate-ILD functions. Related to Figure 7.

Predicted rate-ILD functions were estimated in similar fashion as for the predicted response to the repeated stimulus sequence (Figure 4). (A and D) Predicted (colored lines) and recorded rate-ILD functions (gray, same as Figures 7A and D). The estimated rate-ILD functions' usually closely resemble the recorded functions' shape and show very similar mean-dependent shifts in threshold (B and C) and variance-dependent changes in slope (E and F). However, the predicted rate-ILD functions often exhibited higher overall firing rates because the linear-nonlinear models do not take account of spiking history and therefore predict more sustained spiking output than the neurons are capable of producing in response to the static stimuli. For this reason, the predicted and recorded functions' firing rates plotted in (A) and (D) have been normalized (by the maximum rate of the baseline functions). Furthermore, the linear-nonlinear models for the lowest variance conditions were not capable of predicting rate-ILD functions for the entire range of stimulus values; consequently, the two highest variance functions in (D) remain incomplete and the mean slope (F) is only plotted for three of the four variance conditions.

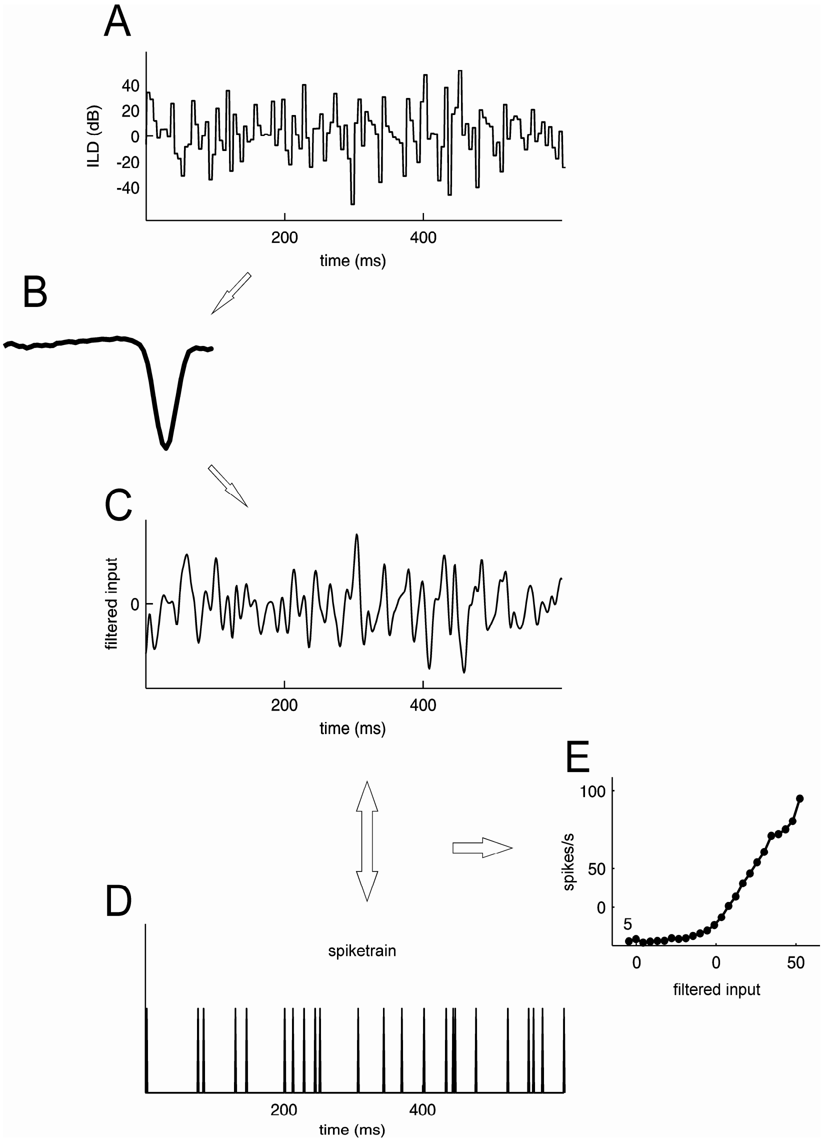

Figure S5. Schematic illustration of linear-nonlinear model analysis. Related to Figure 4.

A neuron's linear filter is obtained by computing the spike triggered average, i.e. calculating the average of all stimulus segments that preceded a spike, and represents the neuron's preferred stimulus feature. Then the stimulus (A) is passed through the filter (B) to obtain the filtered input signal (C). The filtered signal provides a measure of the similarity between the stimulus and the neuron's preferred feature. Then the input-output function (E), which describes the relationship between the filtered input and spike rate and, thus, indicates a neuron's sensitivity to its preferred stimulus feature, is computed. This is done by relating the filtered input (C) to the corresponding spike train (D) so that the average spike rate associated with each value of filtered input can be calculated.
